# Evolution of sex determination in crustaceans

**DOI:** 10.1007/s42995-023-00163-4

**Published:** 2023-02-22

**Authors:** Zhiqiang Ye, Trent Bishop, Yaohai Wang, Ryan Shahriari, Michael Lynch

**Affiliations:** 1 Center for Mechanisms of Evolution, Arizona State University, Tempe, AZ 85287 USA; 2 Institute of Evolution and Marine Biodiversity, KLMME, Ocean University of China, Qingdao, 266003 China

**Keywords:** Crustacean, Sex determination, Sexual differentiation, *Dmrt*, *Daphnia*

## Abstract

Sex determination (SD) involves mechanisms that determine whether an individual will develop into a male, female, or in rare cases, hermaphrodite. Crustaceans harbor extremely diverse SD systems, including hermaphroditism, environmental sex determination (ESD), genetic sex determination (GSD), and cytoplasmic sex determination (e.g., *Wolbachia* controlled SD systems). Such diversity lays the groundwork for researching the evolution of SD in crustaceans, i.e., transitions among different SD systems. However, most previous research has focused on understanding the mechanism of SD within a single lineage or species, overlooking the transition across different SD systems. To help bridge this gap, we summarize the understanding of SD in various clades of crustaceans, and discuss how different SD systems might evolve from one another. Furthermore, we review the genetic basis for transitions between different SD systems (i.e., *Dmrt* genes) and propose the microcrustacean *Daphnia* (clade Branchiopoda) as a model to study the transition from ESD to GSD.

## Introduction

Sex determination (SD) systems designate whether an individual will develop into a male, female, or in rare cases hermaphrodite. One potential advantage of having separate sexes (male and female) is being able to bring beneficial alleles from both individuals together when sexual reproduction occurs (McDonald et al. 2016; Muller 1932; Otto 2009). Of the two main types of SD systems, i.e., genetic sex determination (GSD) and environmental sex determination (ESD), the former is controlled by genetic factors as expansive as sex chromosomes, whereas the latter is driven by environmental cues (e.g., temperature and photoperiod). GSD can be further divided into XY and ZW systems depending on whether the males or females are heterogametic. XY systems are found in many species, such as humans, *Drosophila*, and *C. elegans*, in which the Y chromosome is extremely degenerate or absent (Bachtrog 2013; Blackmon et al. 2017). Contrary to the XY system, under the ZW system, females are heterogametic (ZW) and males are homogametic (ZZ). The ZW system is utilized by birds (Stevens 1997), reptiles (Ezaz et al. 2009), insects (Blackmon et al. 2017), and many crustaceans (Cui et al. 2015; Jiang and Qiu 2013; Parnes et al. 2003).

**Fig. 1:**
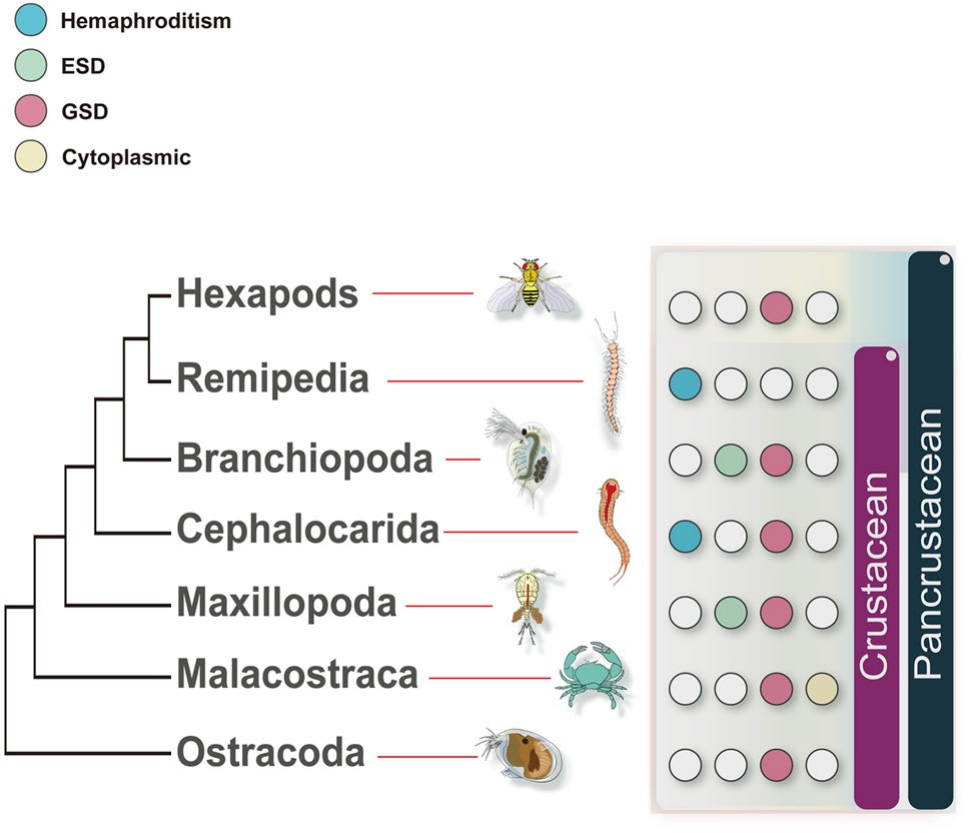
Phylogeny of extant crustaceans and modes of sex determination for each clade. The original phylogenetic tree was generated by Schwentner et al. (2017) based on the PhyloBayes analysis of 1077 decisive orthogroups and 301,748 amino acid positions with the Site-Heterogeneous CAT-GTR Model. We modified the tree to show only the major Pancrustacean clades. *ESD* environmental sex determination, *GSD* genetic sex determination; cytoplasmic (e.g., *Wolbachia* controlled SD system)

Crustaceans originated ~ 500 million years ago during the Precambrian period (Zhang et al. 2007), and now include ~ 67,000 species, such as crabs, lobsters, crayfish, shrimps, and water fleas. Although most crustaceans are aquatic and free-living, some are terrestrial (e.g., woodlice) or parasitic (e.g., fish lice). Crustaceans are phylogenetically close to insects, and together form the Pancrustacean clade (Budd and Telford 2009) (Fig. 1). Extant crustacean lineages can be classified into four primary clades: Branchiopoda (e.g., water fleas; and clams, fairy, and tadpole shrimps), Maxillopoda (e.g., barnacles and copepods), Malacostraca (e.g., shrimps, crabs, lobsters, and crayfish), and Ostracoda (e.g., sea firefly) (Fig. 1), with the number of species ranging from 800 in Branchiopoda to 25,000 in Malacostraca (Schwentner et al. 2017). Also, crustaceans contain two minor clades, Remipedia and Cephalocarida (e.g., horseshoe shrimp), each of which contains 12–17 species (Schwentner et al. 2017).

Crustaceans have diversified SD systems in each clade (Bauer 2000; Becking et al. 2017; Chandler et al. 2018; Hessler et al. 1995; Toyota et al. 2021; Yager 1991). In Malacostraca and Ostracoda, sex is almost exclusively controlled by genetic factors (Becking et al. 2017; Fang et al. 2020; Parnes et al. 2003), whereas sex determination in Maxillopoda and Branchiopoda is largely by environmental factors (Blaxter et al. 1998; Michaud et al. 2004; Toyota et al. 2015a, b). The two most primitive groups, Cephalocarida and Remipedia, reproduce exclusively by simultaneous hermaphroditism (Hessler et al. 1995; Yager 1991).

These highly variable SD systems provide a basis for studying how SD evolves in the diversified crustacean taxa. Moreover, some crustaceans (e.g., *Lysmat*) have unique sexual systems, such as protandric simultaneous hermaphroditism (Bauer 2000), which is important for the study of sex-allocation theory. In addition, transitions from ESD to GSD have occurred in lineages such as *Daphnia* (clade Branchiopoda), providing a valuable model for studying the rapid transition between system types. In this review, we survey our understanding of the SD systems in crustaceans and further discuss their evolutionary paths.

### Sex determination systems in crustaceans

In the largest clade of crustaceans, Malacostraca (> 40,000 species), both ZW and XY SD systems are present. The ZW system is found in most shrimps and crayfishes (Jiang and Qiu 2013; Parnes et al. 2003), whereas the XY system is employed by groups, such as crabs and lobsters (Becking et al. 2017; Chandler et al. 2017; Fang et al. 2020; Mlinarec et al. 2016; Triño et al. 1999) (Table 1). The presence of both XY and ZW systems in species belonging to the same genera of Malacostraca implies the potential for rapid transitions between such systems (Becking et al. 2017). In addition, some species in the Malacostraca (e.g., *Palaemon elegans*) have been found to have multiple sex chromosomes: *X*_1_, *X*_2_, and *Y*, resulting in a SD system of *X*_1_*X*_1_*X*_2_*X*_2_♀/*X*_1_*X*_2_*Y*♂ (Torrecilla et al. 2017). Additionally, in *Armadillidium vulgare* (clade Malacostraca), SD is mediated by bacterial endosymbionts (i.e., *Wolbachia*), which can convert ZZ genetic males to phenotypic females (Cordaux et al. 2011; Rigaud et al. 1997).

**Table 1:** Major modes of sex determination in crustaceans

Taxonomy	Species	Mechanism	References
Branchiopoda	*Eulimnadia texana*	GSD: males and hermaphrodites	Sassaman and Weeks (1993)
	*Daphnia pulex* *Daphnia magna*	ESD and GSD	Toyota et al. (2015a, b); Reisser et al. (2017); Ye et al. (2019) Hobaek and Larsson (1990; Reisser et al. (2017)
Maxillopoda	*Cirripedia thoracica*	GSD	Gomez (1975)
	*Tigriopus californicus*	ESD and GSD	Alexander et al. (2015); Voordouw and Anholt (2002)
	*Pandalus latirostris*	Sequential hermaphroditism	Chiba (2007)
Malacostraca	*Armadillidium vulgare*	ZZ ♂/ZW ♀ and ZZ ♂ + Wolbachia = ♀	Rigaud et al. (1997)
	*Macrobrachium rosenbergii* *Cherax quadricarnatus*	ZZ ♂/ZW ♀	Jiang and Qiu (2013); Parnes et al. (2003)
	*Porcellio dilatatus dilatatus* *Sagmariasus verreauxi* *Austropotamobius pallipes* *Charybdis feriatus*	XY ♂/XX ♀	Becking et al. (2017); Chandler et al. (2017); Fang et al. (2020); Mlinarec et al. (2016); Triño et al. (1999)
	*Palaemon elegans*	*X*_1_*X*_1_*X*_2_*X*_2_♀/*X*_1_*X*_2_*Y*♂	Torrecilla et al. (2017)
Ostracoda	*Pupilometers carcharodonta* *Vargula tsuji* *Euphilomedes* sp. *Euphilomedes morini*	XX ♀/X0 ♂	Jeffery et al. (2017); Sajuthi et al. (2015); Turgeon and Hebert (1995)
	*Cyprinotus incongruens* *Heterocypris incongruens*	*X*_2n_0 ♀/*X*_n_*Y* ♂	Dietz (1957); Havel et al. (1990)

*GSD* genetic sex determination, *ESD* environmental sex determination

Ostracoda is the second-largest clade of crustacean with more than 13,000 living species. In Ostracoda, most studied species have X0 SD system, with sex dimorphism being controlled by the dosage effect of genes on chromosome *X* (Jeffery et al. 2017; Sajuthi et al. 2015; Turgeon and Hebert 1995). Although a few Ostracoda species have a *Y* chromosome, it appears that the sex-determining locus is not located on the *Y*, and sex is determined by the number of *X* chromosomes (Dietz 1957; Havel et al. 1990).

In contrast to Malacostraca and Ostracoda, in which sex is nearly always determined by genetic factors, sex in Maxillopoda and Branchiopoda species is often at least in part influenced by environmental factors (Blaxter et al. 1998; Michaud et al. 2004; Toyota et al. 2015a, b). For example, the copepod *Tigriopus californicus* (clade Maxillopoda) produces more males at higher temperatures (Voordouw and Anholt 2002). Similarly, food concentration and quality determines the percentage of males in other copepods (Irigoien et al. 2000; Michaud et al. 2004). Furthermore, some barnacles and shrimps (clade Maxillopoda) produce male and female gametes at distinct life stages (Chiba 2007; Fukuhara 1999; Subramoniam 2013), which is a system known as sequential hermaphroditism. In *Daphnia* (clade Branchiopoda), females are generally produced under favorable conditions, whereas males are more likely to be produced in unfavorable conditions, such as crowding (Hebert 1978) or short photoperiod (Toyota et al. 2015a, b).

Although most crustaceans have separate sexes, Cephalocarida and Remipedia species are exceptions. They reproduce exclusively by simultaneous hermaphroditism in which individuals have both male and female sex organs, and generate both types of gametes (Hessler et al. 1995; Yager 1991). Simultaneous hermaphroditism has been observed also in a small number of decapod species (clade Malacostraca) (Bauer and Holt 1998; Fiedler 1998). Simultaneous hermaphroditism is not without disadvantages as each parent must grow and maintain two sets of reproductive machinery (Heath 1977). It has been suggested that simultaneous hermaphroditism evolved as a result of the scarcity of mating partners (Cabej 2013). Consistent with this, Remipedia have been reported to reside solely in submerged caves (Yager 1991) and are slow movers (Regier et al. 2010), which may make finding mating partners more challenging.

### Evolution of sex determination in crustaceans

Because Cephalocarida and Remipedia are the most basal crustacean clades (Fig. 1), it has been proposed that they represent ancestral SD system in crustaceans, i.e., simultaneous hermaphroditism (Legrand et al. 1987; Rigaud 1991). A different type of hermaphroditism known as sequential hermaphroditism is more prevalent in crustaceans particularly in barnacles and shrimps (Chiba 2007; Fukuhara 1999; Subramoniam 2013). Species with sequential hermaphroditism create male and female gametes at distinct periods of development, and the two sexes are functionally and morphologically different. In crustaceans, sequential hermaphroditism is an example of phenotypic flexibility in response to environmental changes, supporting the size advantage theory (Warner 1988). Individual crustacea develop in size as they age because they tend to grow continually throughout their lives. According to the size advantage theory, sex change is preferable when the reproductive cost of one sex increases faster than that of the other as body size increases. Size change is employed to optimize the combined fitness of an individual’s male and female stages. Depending on which sex matures first, sequential hermaphroditism is classified as protandry or protogyny. The former is utilized by a vast majority of Malacostraca species (Bauer 1986) in which individuals mature first as males and later change sex to females (Bauer 2000). Females benefit from large size because egg formation demands a significant amount of energy, and the egg survival rate is closely associated with body size in crustaceans (Bauer 2020). Conversely, some isopods (clade Malacostraca) rely on protogyny in which larger males have a reproductive advantage over smaller ones, associated with protection of mates (Abe and Fukuhara 1996). Consistent with the size advantage theory, Nakashima (1987) discovered that *A. dorsalis* competes for females during the breeding season, with larger males being more successful at copulating than smaller males.

Simultaneous hermaphroditism may develop into sequential hermaphroditism if oogenesis is not synchronized with spermatogenesis (Bauer 2000; Hoffman 1972). In some cases, reproductive resources are limited in brooding animals due to a lack of brooding space, and resources may be totally dedicated to sperm production, resulting in sequential hermaphrodites (Heath 1979). Conversely, sequential hermaphrodites could evolve into simultaneous hermaphrodites if both male and female organs are generated concurrently during the female phase (Bauer 2000; Hoffman 1972). This typically happens when population density is low and difficult to find mating partners.

ESD, which is utilized by a large number of crustaceans, shares similar characteristics with sequential hermaphroditism (Breton et al. 2018). First, neither system requires genetic differentiation between the two sexes. Second, both ESD and sequential hermaphroditism typically result in a skewed sex ratio. Third, sex change is preferable in both systems when one sex has a greater reproductive fitness than the other. Fourth, both ESD and sequential hermaphroditism share a common sex determination mechanism—stress-related pathways. Social structure could lead to situations in which individuals within a group experience varying degree of stress. It has been demonstrated that social factors within mating groups influence sex change in crustaceans, such as hippolytid shrimp (Bauer and Baeza 2004; Lin and Zhang 2001) and pandalid shrimp (Carpenter 1978; Charnov and Hannah 2002), both of which are hermaphrodites. Due to these similarities, it has been postulated that ESD may evolve from sequential hermaphroditism by a heterochronic shift (Straková et al. 2020).

GSD, which is the most prevalent form of SD in crustaceans, may evolve from either hermaphroditism or ESD. In the case of hermaphroditism, distinct sexes could arise by male- or female-sterility mutations resulting in a system in which genetic females or males coexist with hermaphrodites (Charlesworth and Charlesworth 1978). Additional mutations beneficial to each sex will be retained in such a system if they are linked to the respective sex-determining gene (Edwards 1998). The sex-determining locus and its associated sex-specific genes could then lead to the establishment of a “proto-sex chromosome”, which may be maintained by chromosomal inversion or located in the centromere region, as both structural features minimize or eliminate recombination (Natri et al. 2019). GSD may also emerge from ESD as a result of a male- or female-sterility mutation, in which some females or males are genetically determined whereas others are subject to ESD. A system of this type could be maintained if it restores the sex ratio or lowers the cost of inbreeding (Edwards 1998; Reisser et al. 2017; Ye et al. 2019). It is believed that the shift from ESD to GSD is unidirectional as the majority of the well-supported transitions have occurred in the direction of ESD to GSD (Straková et al. 2020), with rare instances of GSD to ESD when male heterogamety disappeared (Bull 1981). When the environment is unpredictable or very variable, evolution from ESD to GSD is favored as GSD can maintain balanced sex ratios (Bull 1983). For example, snow skinks used ESD at low elevations, but shift to GSD at higher elevations where fluctuations in temperature are more pronounced (Pen et al. 2010).

**Fig. 2:**
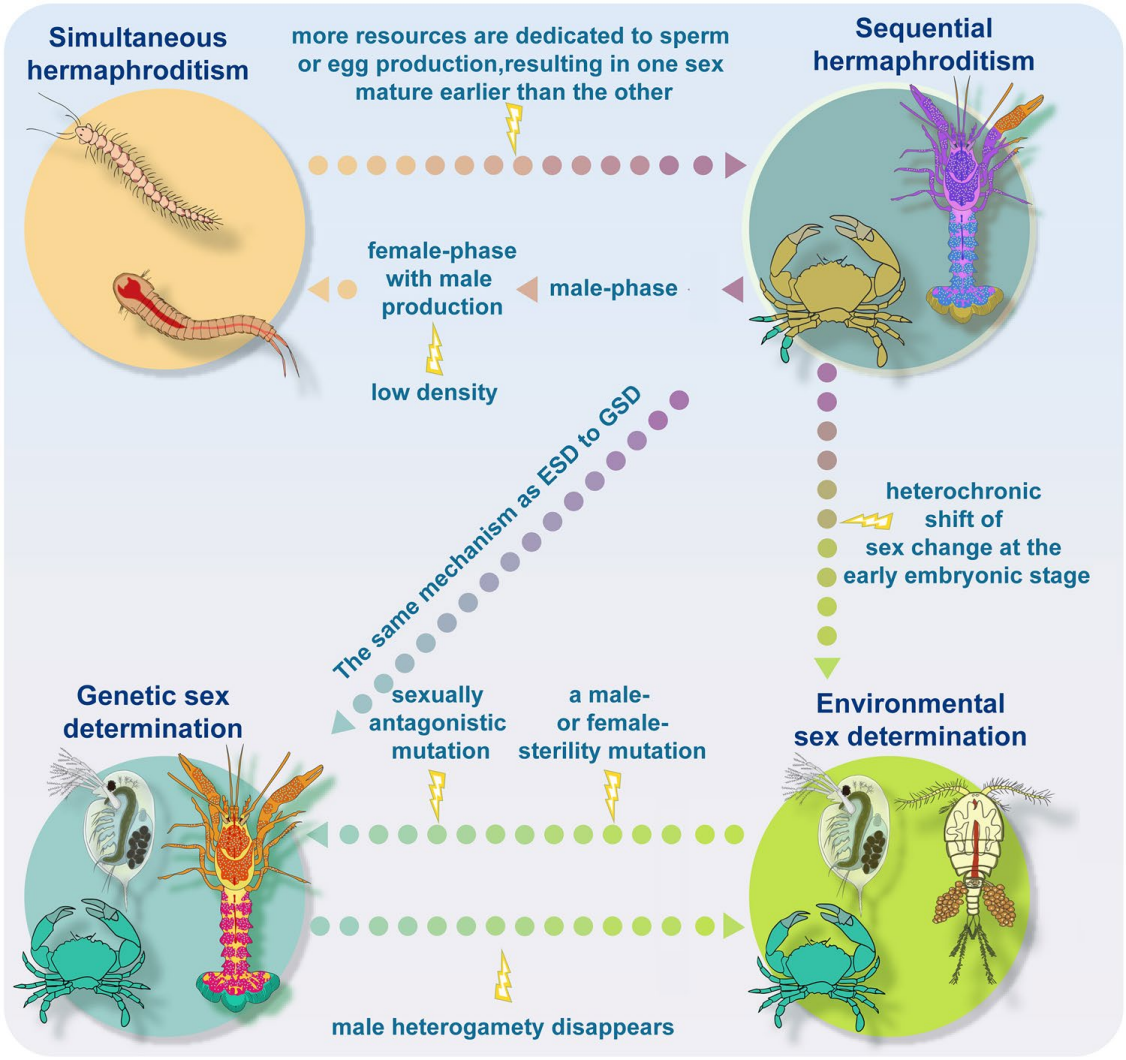
Evolutionary routes of sex determination systems in crustaceans. The flash arrow indicates the trigger required for each transition. *GSD* genetic sex determination, *ES*D environmental sex determination

In summary, while direct evidence for transitions among different SD systems in crustaceans is currently scarce, we infer putative evolutionary routes based on the available research. It has been proposed that crustaceans exhibited ancestral hermaphroditism in the form of simultaneous hermaphroditism and/or sequential hermaphroditism (Hessler et al. 1995; Yager 1991). ESD may have evolved from sequential hermaphroditism by a heterochronic shift of sex change during the early embryonic stage (Straková et al. 2020) (Fig. 2). Both hermaphroditism and ESD have the potential to progress to GSD with rare instances of reversion from GSD to ESD. We summarize the possible evolutionary routes for the SD systems in crustaceans in Fig. 2.

### The role of *Dmrt* genes in sex determination

**Fig. 3:**
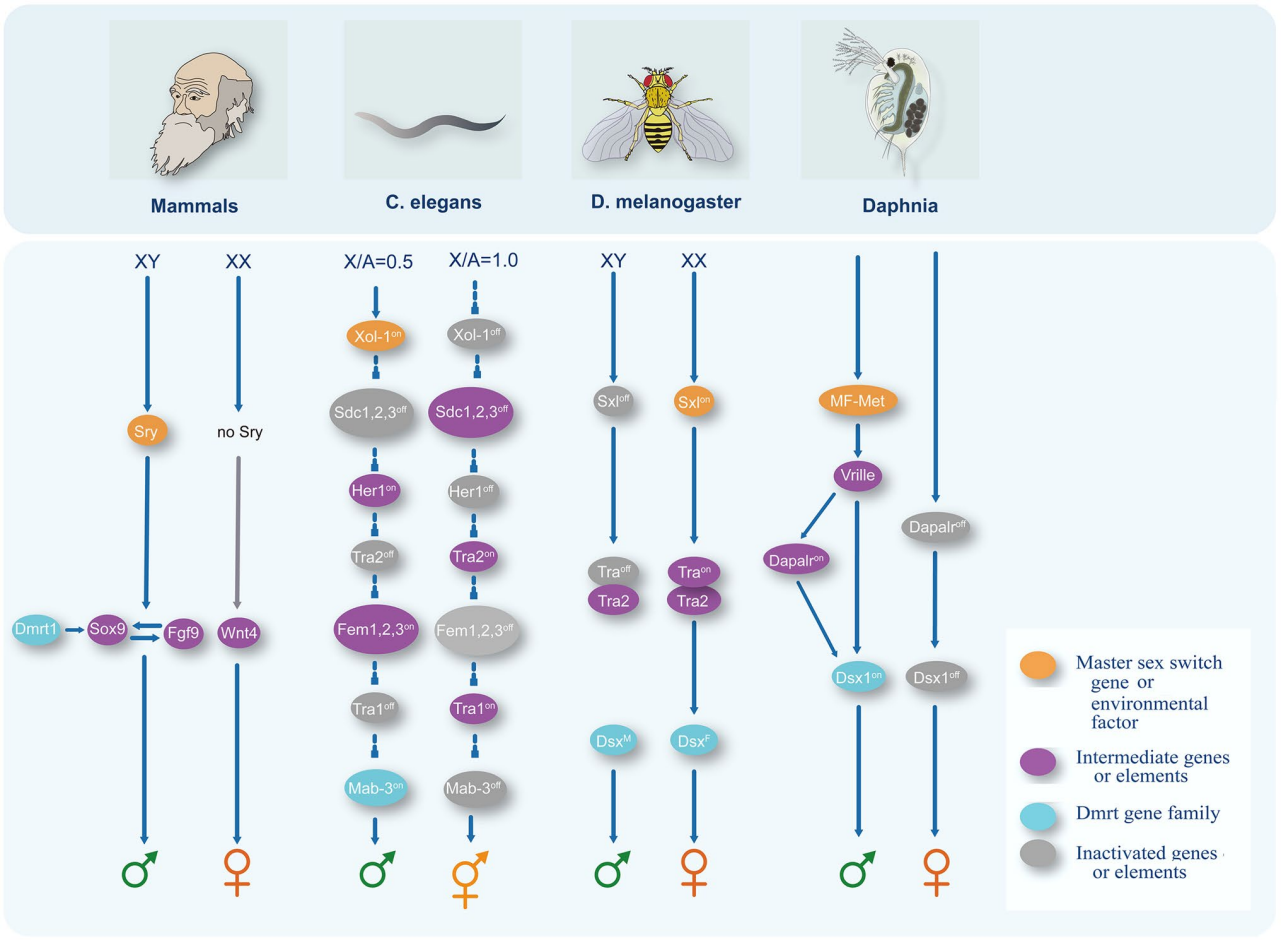
Sex switch and *Dmrt* genes in the sex determination pathways in diverse model species*. DMRT* doublesex and male abnormal-3-related transcription factors gene family, *Sry* sex-determining Region Y, *Sox9* sry-related box 9, *Xol-1* XO lethal protein 1, *Tra* transformer; *Sxl* sex lethal, *MF* methyl farnesoate, *Dapalr* doublesex1 alpha promoter-associated long non-coding RNA, *Mab-3* male abnormal-3. *Wtn4* Wnt family member 4. Arrows indicate positive regulation, and crossbars indicate repressive regulation

As the SD systems in crustaceans are capable of transitioning among each other, it is critical to understand the genetic basis for those transitions. Although the mechanism of sex determination varies across species, the underlying sex regulator genes appear to have converged on the doublesex and male abnormal-3 (*Mab*-*3*)-related transcription factor (*Dmrt*) gene family (Kopp 2012; Zarkower 2001) (Fig. 3). In mammals, development of male embryos is regulated by the Y chromosome-linked *Sry* gene, with *Sry* depletion resulting in ovary development (Sekido and Lovell-Badge 2009; Wilhelm et al. 2007). *Sox9* (*Sry*-related box 9) is a downstream gene of *Sry* that is essential for testis development, and its absence results in male–female sex reversal (Foster et al. 1994). *Dmrt1* is thought to be a pioneer factor for opening chromatin and allowing binding of *Sox9* (Lindeman et al. 2021), and is expressed primarily in the testis following sex differentiation (Kim et al. 2007a, b). Loss of *Dmrt1* function results in decreased *Sox9* expression, which eventually results in sex reversal (Matson et al. 2011). The expression of *Sox9* is maintained by a positive feedback loop with fibroblast growth factor 9 (*Fgf9*) (Piprek 2009), which together activates the male pathway (Kim et al. 2006a, b).

In *C. elegans*, sex is determined in a dose-dependent manner (males have a X chromosome and autosomes ratio of 0.5 (XO), whereas hermaphrodites have a ratio of 1.0 (XX)) by a master switch gene called *Xol-1* (Luz et al. 2003). *Xol-1* promotes male development, and loss-of-function mutations cause lethality in male animals (Miller et al. 1988). High levels of *xol-1* expression in XO animals could repress the activity of *sdc-1*, *sdc-2*, and *sdc-3* genes, whereas low *xol-1* levels in XX animals permits the activation of the three sdc genes (Luz et al. 2003) (Fig. 3). Proteins from all three sdc genes function in suppression of *her-1* (Yonker and Meyer 2003), which is a protein that promotes male development by inhibiting the function of *Tra-2* (Perry et al. 1993; Pilgrim et al. 1995). *Tra-2* inactivates the expression of the three fem (feminization) proteins (Gaudet et al. 1996; Pilgrim et al. 1995), which further inhibits the *tra-1* activity in XO animals (Hodgkin 1986; Kimble et al. 1984). *Mab-3*, which is a member of the *Dmrt* gene family, is a *Tra1* target gene that controls male sexual development and behavior in *C. elegans* (Yi et al. 2000). *Tra1* represses the transcription of *Mab-3* in XX animals resulting in hermaphrodites, where in XO animals *Tra-1* is inactivated and the male fate was determined (Yi et al. 2000).

In *Drosophila*, the ratio of X chromosomes to autosomes determines whether the Sex lethal (*Sxl*) gene is on (in XX females) or off (in XY males) (Fig. 3). *Sxl* regulates the production of *Tra* protein in females (Salz and Erickson 2010). In males, due to the lack of *Sxl*, an mRNA with no long open reading frame is produced and functional protein is not generated (Fig. 3). *Tra* then controls sex-specific splicing of *doublesex* (*Dsx*) pre-mRNA (Bachtrog et al. 2014). *Dsx* was the first *Dmrt* gene discovered in insects, and its involvement in sex determination has been established (Burtis et al. 1991). The male and female-specific *Dsx* proteins then determine the unique somatic structures and external morphology in each sex (Baker et al. 2001; MacDougall et al. 1995).

*Dmrt* is also a critical sex determinant in crustaceans, such as *D. magna* (Kato et al. 2011a, b) and *D. pulex* (Xu et al. 2014). Male production is stimulated in *D. magna* by the hormone methyl farnesoate (MF) (Toyota et al. 2015a, b), which is then directly coupled to the methoprene-tolerant (Met) and steroid receptor coactivator (SRC) complex (Miyakawa et al. 2013). The binding sites of Met have been found in a bZIP transcription factor, *Vrille* (Mohamad Ishak et al. 2017), suggesting that the MF–met complex may directly activate *Vrille* (Fig. 3). *Vrille* then regulates the expression of *Dsx1* and doublesex1 alpha promoter-associated long non-coding RNA (*DAPALR*) (Kato and Watanabe 2022) (Fig. 3). In addition, *DAPALR* could regulate *Dsx1* expression in *trans* (Kato et al. 2018). *Dsx1* is exclusively expressed in male *Daphnia* where its transcripts are found largely in male-specific structures (Kato et al. 2011a, b). Reduced *Dsx1* expression in *D. magna* causes testes to develop an ovary-like morphology, and ectopic *Dsx1* expression in female embryos leads to the development of male-like phenotypes (Kato et al. 2011a, b). Additionally, *Dmrts* have been identified in other crustaceans, such as Penaeidae (prawns), Palinuridae (lobsters), Palaemonidae (shrimp) and Portunidae (crabs) (Chandler et al. 2016). Moreover, their functional conservation has been demonstrated in crustaceans, such as the eastern spiny lobster (*S. verreauxi*) (Chandler et al. 2017).

The conservation of *Dmrt* genes in regulating SD allows for the study of SD evolution in crustaceans, particularly in species with little genetic background. First, *Dmrt* genes share a common function in sex determination and sex differentiation across taxa (Kopp 2012), which is to promote male-specific development and differentiation (Balciuniene et al. 2006; Kim et al. 2007a, b). This function is conserved in species with ESD (e.g., turtles) and GSD (e.g., *Drosophila*, mammals) systems (Kopp 2012). Second, *Dmrt* genes are expressed exclusively in developing gonads of all animals including mammals (Kim et al. 2003; Raymond et al. 2000), flies (Hempel and Oliver 2007), nematodes (Yi et al. 2000) and crustaceans (Farazmand et al. 2010; Kato et al. 2011a, b; Zhang and Qiu 2010). Third, *Dmrt* genes share a common DNA-binding domain (DM domain) that is highly conserved across phyla (Chandler et al. 2018; Raymond et al. 1998), whereas there is substantial sequence variation outside of the DM domain, sequences within the DM domain are extremely conserved (Chandler et al. 2018). The conserved nature of the DM domain enables the identification of genes that bind to it. Indeed, numerous genes associated with sex determination have been found in humans (Murphy et al. 2010) and *Drosophila* (Luo et al. 2011) via their physical or structural association with the DM domain. However, such studies are currently scarce in crustaceans.

Regardless of the conservation, the mechanisms by which *Dmrt* influences sex determination in each species may vary. For example, in insects, different isoforms of *Dsx* derived by alternative splicing are expressed in males and females, whereas in *Daphnia*, *Dsx* is expressed exclusively during male development (Kato et al. 2011a, b). It is worth mentioning that whereas *Dmrt* genes are not necessarily the master sex-determining genes, they often directly or indirectly interact with the master switch gene. Thus, identifying *Dmrt* genes will always be helpful in locating the SD locus and/or mechanisms in crustaceans.

### Using *Daphnia* as a model to study the evolution of sex determination

Transitional species are of particular interest for shedding light on the evolution of sexual determination. Members of the genus *Daphnia* are now experiencing such a transition. Most *Daphnia* reproduce by cyclical parthenogenesis, with extended periods of parthenogenesis interspersed with sexual resting-egg production, generally on a yearly cycle (Hebert 1978). Parthenogenetic eggs may develop into females or males as sex determination is typically induced by environmental factors (e.g., short photoperiod). However, in some *Daphnia* populations, sex is also controlled by genetic factors (Reisser et al. 2017; Ye et al. 2019). Within such *Daphnia* populations, some females have lost the ability to produce males, resulting in the formation of non-male-producing (NMP) clones (Galimov et al. 2011; Tessier and Cáceres 2004). The coexistence of NMP clones and hermaphrodites (MP clones) in *Daphnia* creates a system called gynodioecy in which only females are genetically determined.

Because crosses between NMP and MP clones consistently produce a close to 1:1 ratio of NMP and MP offspring, whereas almost all offspring of MP × MP crosses exhibit MP phenotypes (Galimov et al. 2011; Innes and Dunbrack 1993), the presence of a dominant allele at a single locus underlying the NMP phenotype appears likely. To be more precise, all NMP clones are hypothesized to be WZ heterozygous at the locus conferring the NMP phenotype, whereas MP clones are thought to be ZZ homozygotes as is the case with classical W/Z sex determination systems. Additionally, it has been reported that the NMP phenotype is regulated by a single dominant allele contained within a 1.2 Mb non-recombining region in *D. pulex* (Ye et al. 2019) although it is largely unknown which genes within this region are involved in SD. The functional conservation of the *Dmrt* genes provides a good opportunity to connect genes in the 1.2 Mb region to the master sex switch gene (i.e., *Dsx*) in *Daphnia*.

To identify potential genes implicated in the shift from ESD to GSD, it is necessary to first understand the SD pathway in *Daphnia*. Typically, SD in *Daphnia* is regulated via environmental cues, such as short photoperiod or by adding exogenous juvenile hormone (JH). JH is an essential endocrine factor that regulates molting and metamorphosis in insects (Nijhout 1998). JH was found in Malacostraca (e.g., crab and crayfish) and Branchiopoda (e.g., *Daphnia*) species, but JH in those species lacks the epoxide group compared to that in insects (Laufer et al. 1987). It has been suggested that the JH in crustaceans, methyl farnesoate (MF), is important in molting and reproduction (Homola and Chang 1997). Additionally, JH has been shown to induce male production in *Daphnia* (Abe et al. 2015) and other cladoceran (clade Branchiopoda) species, such as *Moina, Ceriodaphnia*, and *Bosmina* (Kim et al. 2006a, b; Oda et al. 2005). Thus, it has been postulated that JH plays a general role in SD throughout the clade Branchiopoda (Kim et al. 2006a, b; Olmstead and Leblanc 2002).

**Fig. 4:**
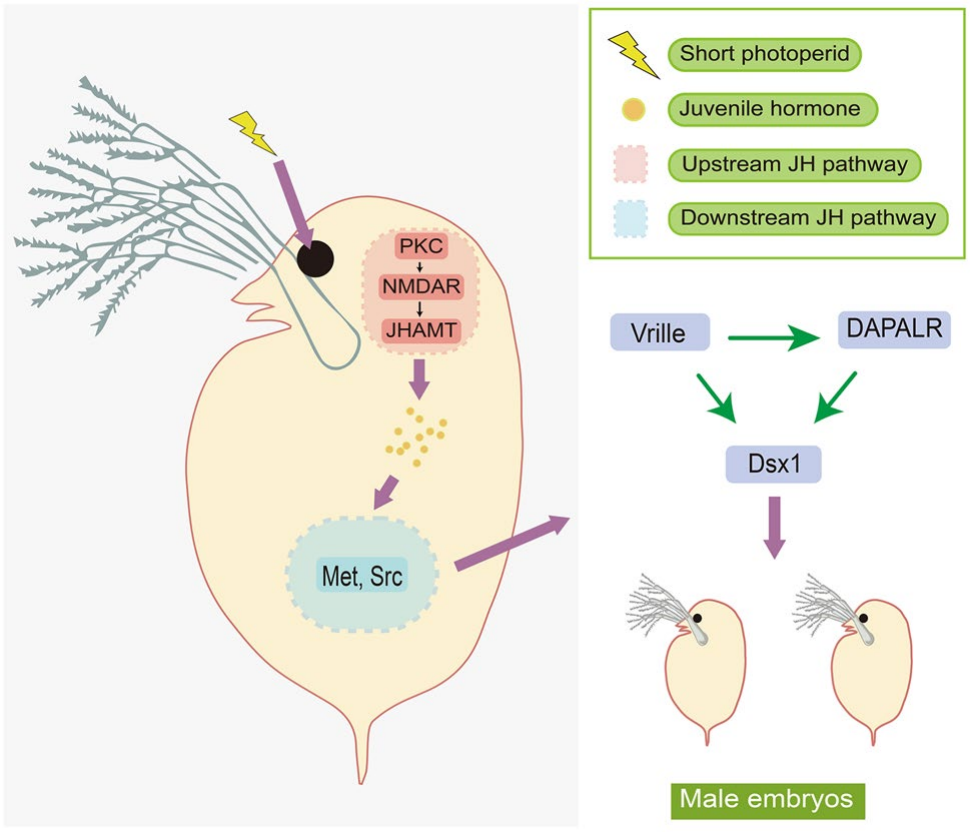
Signaling cascades of the sex determination pathway in *Daphnia* (Branchiopoda). *JH* juvenile hormone, *PKC* protein kinase C, *NMDAR* N-methyl-D-aspartic acid receptor, *JHAMT* juvenile hormone acid O-methyltransferase, *Met* methoprene-tolerant, *SRC* steroid receptor coactivator, *DAPALR* doublesex1 alpha promoter-associated long non-coding RNA, *Dsx* doublesex. Regulation of DAPALR, Vrille, Dsx1 happened in embryos

In *Daphnia*, the SD pathway could be separated into two parts, namely the upstream and the downstream JH pathway (Fig. 4). The signal (e.g., short photoperiod) from the environment activates protein kinase C (PKC) in the upstream JH pathway. Then, PKC promotes the opening of the N-methyl-D-aspartic acid receptor (NMDAR) channel (Toyota et al. 2017) (Fig. 4). NMDAR is a type of ionotropic glutamate receptor that is required for male reproduction, and is believed to act as an upstream regulator of juvenile hormone acid O-methyltransferase (JHAMT) (Toyota et al. 2015a, b). JHAMT is utilized to synthesize JH from farnesoic acid in *Daphnia* (Toyota et al. 2015a, b), and it expresses at a higher level in male-producing conditions than in female-producing ones (Toyota et al. 2015a, b). JH was directly coupled to the methoprene-tolerant (Met) and steroid receptor coactivator (SRC) complex following its synthesis, and mutations within *Daphnia* Met were found to significantly alter the receptor's responsiveness (Miyakawa et al. 2013). Met's immediate target in insects is Krüppel homolog 1 (*kr-h1*) (Cui et al. 2014), but its function in *Daphnia* is still unknown (Toyota et al. 2018). In both *D. magna* and *D. pulex*, the sexual differentiation is eventually mediated by the *Dsx1* gene (Kato et al. 2011a, b; Xu et al. 2014). Kato et al. discovered that the expression pattern for *Dsx1* is male-specific in *Daphnia*, and that knocking down *Dsx1* in male embryos or ectopic expression of *Dsx1* in female embryos led to sex reversed traits (Kato et al. 2011a, b; Toyota et al. 2013). *Dsx1* has been shown to be directly regulated by a bZIP transcription factor, *Vrille* (Mohamad Ishak et al. 2017), as well as by the doublesex1 alpha promoter-associated long non-coding RNA (*DAPALR*) (Kato et al. 2018) (Fig. 4).

The transition from ESD to GSD in *D. pulex* is thought to be induced by a factor downstream of the JH signaling pathway as the phenotype cannot be recovered by exogenous JH (Ye et al. 2019). Notably, a 1.2 Mb region containing 87 genes in NMP clones possesses NMP-specific substitutions, establishing the foundation for further investigation of the genetic components underlying the NMP trait. As a start, it is necessary to determine whether any of the 87 genes are direct targets of Met or Src. Additionally, the 1.2 Mb region in NMP clones may constitute a “proto-sex chromosome”, a hypothesis that may be validated by investigating whether the 87 genes in the NMP region express in a sex-specific manner. Additionally, if the SD transition involves the full 1.2 Mb non-recombining region, it will be worthwhile to determine whether this region is preserved by genome inversion. Finally, elucidating the SD pathway in *Daphnia* will provide an opportunity to investigate the transition from ESD to GSD in crustaceans.

### Future perspectives

In this review, we summarize knowledge about the SD systems in crustaceans and discussed how these distinct systems might evolve from one another. However, due to the lack of research, genes in the SD pathway remain largely unknown in most crustaceans. Up till now, all ideas regarding SD transitions (e.g., from ESD to GSD) have been purely theoretical and require further validation. *Daphnia* could be an excellent model for studying the evolution of SD systems in crustaceans (and more broadly, arthropods) given its high-quality reference genomes, extensive research on the SD pathway, and powerful genetic editing tools such as CRISPR/Cas9. To begin, we could use Chip-seq data to identify genes that interact with *Dsx1*; second, transcriptomic data could be used to validate sex-specific genes in the NMP region; and third, we could compare high-quality genome assemblies from MP and NMP *Daphnia* clones to determine if structural variation (e.g., genome inversion) underlies the transition from ESD to GSD. Along with transitions between SD systems, transitions within the same SD system (e.g., XY to ZW) are widespread among crustacean genera (Becking et al. 2017). These transitions are particularly interesting because they provide insight into the rapid turnover within the same SD system. As such, new investigations aimed at identifying *Dmrt* genes, their targets, and genes that interact with the DM domain in genera with such transitions would be particularly exciting.

## Data Availability

N/A.
